# Longitudinal Multi-Channel Focused Vortex and Vector Beams Generation by Quarter-Wave Plate Meta-Atom Metasurfaces

**DOI:** 10.3390/nano15050324

**Published:** 2025-02-20

**Authors:** Teng Ma, Kaixin Zhao, Manna Gu, Haoyan Zhou, Chunxiang Liu, Chuanfu Cheng, Qingrui Dong, Li Ma

**Affiliations:** 1 School of Physics and Electronics, Shandong Normal University, Jinan 250358, China; mateng_mt1320@163.com (T.M.); zhaokaixin_1999@163.com (K.Z.); gumanna1996@outlook.com (M.G.); zhouhaoyanrock@163.com (H.Z.); liuchunxiang@sdnu.edu.cn (C.L.); chengchuanfu@sdnu.edu.cn (C.C.); 2 Department of Physics, Changzhi University, Changzhi 046011, China

**Keywords:** metasurface, vortex and vector beams, longitudinal multi-channel, quarter-wave plate

## Abstract

Metasurface-based longitudinal modulation introduces the propagation distance as a new degree of freedom, extending the light modulation with metasurfaces from 2D to 3D space. However, relevant longitudinal studies have been constrained to designing the metasurface of half-wave plate (HWP) meta-atoms and generating either non-focused or two-channel vortex and vector beams. In this study, we propose a metasurface composed of quarter-wave plate (QWP) meta-atoms to generate the longitudinal multi-channel focused vortex and vector beams. The metasurface consists of two interleaved sub-metasurfaces of QWP meta-atoms. For each sub-metasurface, the helical and hyperbolic phase profiles are designed independently in the propagation and geometric phases to generate focused co- and cross-polarized vortices with corresponding topological charges. Under the illumination of *x*-linearly polarized light, the metasurface generates two circularly polarized vortices, two linearly polarized vortices, and one vector beam on five focal planes. Theoretical analysis and simulation results demonstrate the feasibility of the proposed QWP metasurface. Our study presents a significant advancement in the development of integrated and multifunctional optical devices and systems, with significant potential applications in light–matter interaction, laser processing, and optical communication.

## 1. Introduction

Vortex beams exhibit a special topological texture in electromagnetic fields, characterized by a zero-intensity singularity and a spiral phase front that carries orbital angular momentum (OAM) [1]. Owing to the OAM, which provides an extra degree of freedom, vortex beams have attracted significant attention in a wide range of research fields, including optical tweezers [2,3], optical detection [4], microscopy and imaging [5], and optical communication [6]. Based on their unique characteristics and broad applications, vortex beams have been developed to vector beams. Vector beams can be generated by superposing two orthogonal circularly polarized vortices and presented by high-order Poincaré (HOP) spheres [7], emerging as a significant research area in optics. The above applications and development are both based on the generation and manipulation of vortex and vector beams. Conventional generation methods include spatial light modulators [8], spiral phase plates [9], and digital micro-mirror devices [10]. However, these optical elements are typically bulky, and the optical paths are complicated. The emergence of metasurfaces has enabled the generation of vortex and vector beams through miniaturized and integrated devices.

Metasurfaces are 2D platforms consisting of sub-wavelength dielectric or metallic meta-atoms [11]. Most of the initial research in metasurfaces has primarily focused on achieving specific functions, such as metalenses [12], polarization beam splitters [13], and polarization converters [14]. Compared to conventional metallic meta-atom metasurfaces, dielectric meta-atom metasurfaces offer an additional degree of freedom for the manipulation of amplitude, phase, and polarization of the optical field by adjusting the geometries in addition to the orientation angles of meta-atoms. Owing to their outstanding capabilities, metasurfaces have found applications in multiphoton interference [15], holographic display and encryption [16], and the generation and manipulation of vortex and vector beams [17,18,19,20,21,22,23,24,25]. These studies utilized half-wave plate (HWP) meta-atoms, which manipulated the output field with only cross-polarized components. However, metasurfaces with quarter-wave plates (QWP) and arbitrary waveplate meta-atoms can simultaneously control both co- and cross-polarized components, offering enhanced design flexibility and expanded functionality in wave manipulation. In the microwave and terahertz bands, a large number of works on QWP metasurfaces have been carried out [26,27,28,29,30,31,32]. In the optical band, QWP meta-atom metasurfaces have also been designed for multifunctional applications. He et al. and Deng et al. [33,34] designed QWP meta-atom metasurface for polarization conversion and wavefront shaping. Badloe et al. [35] and Zhang et al. [36] designed QWP meta-atom metasurfaces with hyperbolic propagation and gradient geometric phase profiles, realizing the multi-channel generation of vortex and vector beams, respectively. Nevertheless, the above studies remain fundamentally constrained to single transverse plane, without considering multiple planes along the propagation direction.

Longitudinal modulation introduces the propagation distance as a new degree of freedom, thereby extending the modulation of the light field from 2D to 3D space. This advancement significantly broadens applications in optical communication [37], laser engineering [38], and light–matter interaction in 3D space. Currently, extensive work has been carried out on manipulating the electromagnetic fields by varying the propagation distance. In relevant longitudinal studies, Yang et al. [39] employed HWP meta-atom metasurfaces to generate zero-order Bessel beams with polarization states rotating along the propagation direction, featuring axicon-phase profiles in the optical band. In the infrared band, Yang et al. [40] realized longitudinal modulation of a linearly polarized vector beam on the equator of a HOP sphere using the finite-difference time-domain (FDTD) method. This was accomplished by applying different axicon phases to the LCP and RCP vortices in the transmitted light field of HWP meta-atom metasurfaces, resulting in a continuous phase difference between the two vortices as the propagation distance increases. In addition, Zheng et al. [41] generated longitudinally varying vector vortex beams through the superposition of two orthogonal circularly polarized vortices with opposite topological charges. Luo et al. [42] enabled a dual-channel transformation from scalar to vector beams along the optical path. All of them designed HWP meta-atom metasurfaces using axilens-phase profiles in the terahertz band. Though adjusting longitudinal distance has been used in metasurface design, it is limited to designing the metasurface of HWP meta-atoms and to generating non-focused or two-channel vortex and vector beams. Metasurfaces composed of QWP meta-atoms capable of generating longitudinal multi-channel focused vortex and vector beams remain unexplored.

In this study, we designed a dielectric QWP metasurface sample to generate longitudinal multi-channel focused vortex and vector beams in the optical band. Here, the focused vortex beams have spatially invariant polarizations, whereas vector beams possess spatially varying polarization. The metasurface consists of two interleaved sub-metasurfaces composed of QWP meta-atoms. The phases of the light field are modulated through a combination of the propagation and geometric phases of sub-metasurfaces. For each sub-metasurface, the propagation phase is designed to contain a helical *φ*_ph, A/B_ and a hyperbolic phase *φ_f_*_2/4_, associated with the topological charges *l*_p, A/B_ and focal length *f*_2/4_, respectively, for vortex generation and beam focusing. The geometric phase is designed to contain a helical phase *φ*_gh, A/B_ with the topological charges *l*_g, A/B_ and a hyperbolic phase *φ_f_*_1/3_ − *φ_f_*_2/4_ for vortex generation and beam focusing. With the left circularly polarized (LCP) light illumination, the co- and cross-components of two sub-metasurfaces are focused on the planes of *z*_2/4_ = *f*_2/4_ and *z*_1/3_ = *f*_1/3_ with the topological charges *l*_p, A/B_ and *l*_p, A/B_ + *l*_g, A/B_, respectively. Under the right circularly polarized (RCP) light illumination, the co- and cross-components are focused on the planes of *z*_2/4_ = *f*_2/4_ and *z*_3/5_ = *f*_3/5_ with the topological charges *l*_p, A/B_ and *l*_p, A/B_ − *l*_g, A/B_, respectively. The conditions of *φ_f_*_3_ = 2*φ_f_*_2_ − *φ_f_*_1_ and *φ_f_*_5_ = 2*φ_f_*_4_ − *φ_f_*_3_ are satisfied, with the constraint of *l*_p, B_ + *l*_g, B_ = − (*l*_p, A_ − *l*_g, A_) holding as well. Therefore, two circularly polarized vortex beams, two linearly polarized vortex beams, and one vector beam are generated on the corresponding focal planes with *x*-linearly polarized (*x*-LP) light illumination, which contains the LCP and RCP components. We begin with the theoretical analysis of the generation of longitudinal multi-channel focused vortex and vector beams using a QWP meta-atom metasurface. Subsequently, we simulated the output field of the designed metasurface in the FDTD method. In our design, by combing propagation and geometric hyperbolic phase profiles, each sub-metasurface enables the production of three distinct longitudinal channels under *x*-LP light illumination. The coherent superposition of the dual phase modulations, implemented via an interleaved QWP meta-atom metasurface structure, allows the simultaneous generation of vortex and vector beams across multiple propagation planes. Our study expands the light field manipulation from 2D to 3D, promoting the development of miniaturized and multifunctional optical devices. This is especially relevant for applications involving vortex and vector beams, particularly in the fields of light–matter interaction and optical communication.

## 2. Design and Method

### 2.1. Overview of Principle

**Figure 1:**
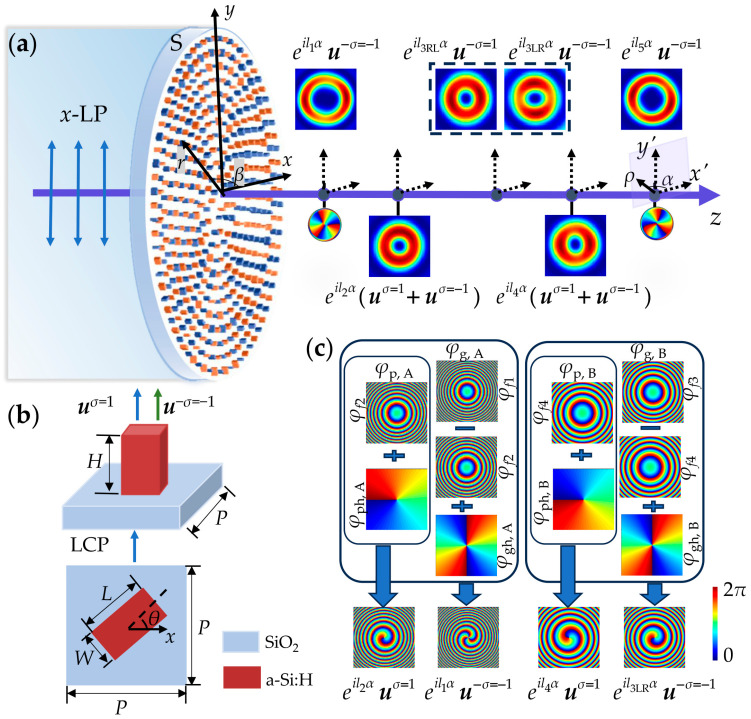
(**a**) Schematic of generating longitudinal multi-channel vortex and vector beams using QWP meta-atom metasurfaces under *x*-LP illumination. *r* and *β* are the radial coordinate and polar angle of the metasurface plane, respectivley. The dotted arrows indicate the axes of the observation planes, with *ρ* and *α* representing the radial coordinates and polar angles, respectivley. The conditions of *l*_1_ = *l*_p, A_ +*l*_g, A_, *l*_2_ = *l*_p, A_, *l*_3RL_ = *l*_p, A_−*l*_g, A_, *l*_3LR_ = *l*_p, B_ +*l*_g, B_, *l*_4_ = *l*_p, B_, and *l*_5_ = *l*_p, B_−*l*_g, B_ are applied. (**b**) The top and side views of a meta-atom, along with the transmitted light field of a meta-atom under LCP light illumination, where *H* = 480 nm, *P* = 380 nm, and *L* and *W* are both in the range of 80 nm to 330 nm. (**c**) Schematic of the propagation and geometric phase profiles of each sub-metasurface in boxes, and the phase profiles of the output light field with LCP light illumination at the bottom.

Figure 1a schematically illustrates the generation of longitudinal multi-channel vortex and vector beams using QWP meta-atom metasurfaces, named as S. The structure S consists of two interleaved sub-metasurfaces, S_A_ and S_B_, denoted as S = S_A_ + S_B_, with the subscripts A and B being used to distinguish between them, as shown in the following description. The top and side views of a meta-atom are shown in Figure 1b. Each sub-metasurface S_A/B_ comprises QWP meta-atoms arranged on concentric circular rings, with the radius being incremented by the lattice period *P*. Along each ring, meta-atoms A and B alternate with separation *P* on the perimeter. This arrangement is basically consistent with the orthogonal square grid of side length *P*. Parameters *H* and *P* represent the height and lattice period, respectively, which remain fixed for meta-atoms A and B. Moreover, *L*, *W,* and *θ* indicate the length, width, and orientation angle of the meta-atom, respectively, which are variable according to the required pointwise propagation and geometric phases for A and B. Under a single circularly polarized light illumination, the output linearly polarized field of a single meta-atom includes the co-polarized component with the same chirality as the incident light and the cross-polarized component with opposite chirality. The polarization direction of this linearly polarized light is determined by the orientation angle *θ* of QWP meta-atom, while the polarization direction determines the phase difference between the two circularly polarized lights, i.e., the geometric phase of the cross-polarized light. Therefore, the orientation angle determines the geometric phase, which is also the phase difference. The incident light can be decomposed into a superposition of CP components to analyze the co- and cross-polarized components of the output light field. The *x*-LP illumination light contains LCP and RCP components, with the unit vectors of ***u****^σ^*^=1^ = [1 *σi*]*^T^* = [1 *i*]*^T^* and ***u****^σ^*^=−1^ = [1 − *i*]*^T^*, respectively, where *σ* = ±1 denotes the chirality. This results in four components in the output field of a single sub-metasurface, S_A_ or S_B_. Specifically, the first one is the co-polarized RCP light, with unit vector ***u****^σ^* ^= −1^; the second one is the cross-polarized LCP component, with unit vector ***u***^−*σ* = 1^, where –*σ* denotes the chirality of the cross-polarized component, under RCP light illumination; the third one is co-polarized LCP light, with unit vector ***u****^σ^*^=1^; and the fourth one is the cross-polarized RCP component, with unit vector ***u***^−*σ*=−1^, under LCP light illumination. These components are marked in order as RRCP_A/B_, RLCP_A/B_, LLCP_A/B_, and LRCP_A/B_ and are focused on the desired observation planes. The propagation distances of these planes are represented by *z_j_* = *f_j_*, where *j* = 1, 2…, 5, corresponding to the five points on the *z*-axis in Figure 1a, respectively, with *f*_1_ < *f*_2_ < *f*_3_ < *f*_4_ < *f*_5_. In our design, RRCP_A_ and LLCP_A_, both with the same topological charge *l*_p, A_, are focused on the focal plane *z*_2_ = *f*_2_, while RRCP_B_ and LLCP_B_, both with the same topological charge *l*_p, B_, are focused on the focal plane *z*_4_ = *f*_4_. These result in two linearly polarized vortex beams, as shown in the total intensity images below the *z*-axis in Figure 1a. LRCP_A_, with topological charge *l*_p, A_ + *l*_g, A_, is focused on the focal plane *z*_1_ = *f*_1_, while RLCP_A_ and LRCP_B_, with topological charges *l*_p, A_ − *l*_g, A_ and *l*_p, B_ + *l*_g, B_, are focused on the focal plane *z*_3_ = *f*_3_. When *l*_p, A_ − *l*_g, A_ = −(*l*_p, B_ + *l*_g, B_), the vector beam is generated on this plane. Meanwhile, RLCP_B_, with topological charge *l*_p, B_ − *l*_g, B_, is focused on the focal plane *z*_5_ = *f*_5_. The circular polarization components’ intensity images of these beams are shown above the *z*-axis in Figure 1a, while the phase images of the beams on the focal planes *z*_1_ and *z*_5_ are displayed below the *z*-axis. In addition, the conditions of *l*_1_ = *l*_p, A_ +*l*_g, A_, *l*_2_ = *l*_p, A_, *l*_3RL_ = *l*_p, A_−*l*_g, A_, *l*_3LR_ = *l*_p, B_ +*l*_g, B_, *l*_4_ = l_p, B_, and *l*_5_ = *l*_p, B_−*l*_g, B_ are also shown in Figure 1a for clarity. In conclusion, two circularly polarized vortices, two linearly polarized vortices, and one vector beam are generated on five distinct focal planes corresponding to different propagation distances *z_j_*, respectively.

### 2.2. Design of Propagation and Geometric Phase Profiles for Sub-Metasurfaces

In order to realize the generation of the above longitudinal multi-channel vortex and vector beams, we further describe the desired phase profiles for sub-metasurfaces based on QWP meta-atoms. The propagation phase profiles are imparted to the co-polarized component, while the combination of the propagation and geometric phase profiles are imparted to the cross-polarized component, with the geometric phase profiles related to the chirality of the incident light. In the following subsection, we first describe the propagation and geometric phases of S_A/B_. The helical phase profile, *φ*_ph, A/B_ (*β*) = *l*_p, A/B_ *β*, is constructed with propagation phase *φ*_p, A/B_ (*r*, *β*, 0), where (*r*, *β*) represents the polar coordinates on the metasurface plane of *z* = 0; *l*_p, A/B_ is the topological charge; and subscripts p and h signify the propagation and helical phases, respectively. Then, the hyperbolic phase profile, $\varphi_{f2/4}(r)=k(f_{2/4}-\sqrt{r^{2}+f_{2/4}^{2}})$, where *f*_2/4_ indicates that the focal lengths are *f*_2_ or *f*_4_, is constructed for beam focusing. *k* = 2*π/λ* is the wavevector. The geometric phase, *φ*_g, A/B_ (*r*, *β*, 0), is used to set up the helical and hyperbolic phase profiles, defined as *φ*_gh, A/B_ (*β*) = *l*_g, A/B_ *β* and *φ_f_*_1/3_ − *φ_f_*_2/4_. Therefore, the propagation and geometric phase of S_A/B_ are as follows:

(1) $$\begin{array}{ll} \varphi_{p,\;A/B}(r,\;\beta ,\;0) & =\varphi_{\mathrm{ph},\;A/B}(\beta )+\varphi_{f2/4}(r)\\ & =l_{p,\;A/B}\beta +k(f_{2/4}-\sqrt{r^{2}+f_{2/4}^{2}}) \end{array},$$

(2) $$\begin{array}{ll} \varphi_{g,\;A/B}(r,\;\beta ,\;0) & =2\sigma \theta_{A/B}(r,\;\beta ,\;0)\\ & =\sigma [\varphi_{\mathrm{gh},\;A/B}(\beta )+\varphi_{f1/3}(r)-\varphi_{f2/4}(r)+2\theta_{0}]\\ & =\sigma [l_{g,\;A/B}\beta +k(f_{1/3}-\sqrt{r^{2}+f_{1/3}^{2}})-k(f_{2/4}-\sqrt{r^{2}+f_{2/4}^{2}})+2\theta_{0}] \end{array},$$

where *θ*_0_ denotes the initial orientation angle of the meta-atom. It is noted that the orientation angle directly governs the geometric phase profile through relation *φ*_g_ = 2*σθ*. The orientation angles of meta-atoms within sub-metasurfaces S_A/B_ are spatially varying so that the orientation angle *θ*_A/B_ of meta-atom A or B is expressed as a function of its position (*r*, *β*, 0), i.e., *θ*_A/B_ = *θ*_A/B_(*r*, *β*, 0). In Figure 1c, the propagation and geometric phases of S_A/B_ are shown with *σ* = 1. Therefore, under the illumination of LCP light, the co-polarized (LLCP_A/B_) vortex in the output light field is focused on the plane of *z*_2/4,_ with the topological charge *l*_p, A/B_, while the cross-polarized (LRCP_A/B_) vortex is focused on the plane of *z*_1/3,_ with the topological charge *l*_p, A/B_ +*l*_g, A/B_, where $\varphi_{f3}=2\varphi_{f1}-\varphi_{f2}$. Similarly, with RCP light illumination, the co-polarized (RRCP_A/B_) vortex is focused on the plane of *z*_2/4_, with the topological charge *l*_p, A/B_. Considering that the sign of *φ*_g, A/B_ changes, the cross-polarized (RLCP_A/B_) vortex is focused on the plane of *z*_3/5_, with the topological charge *l*_p, A/B_ −*l*_g, A/B_, where $\varphi_{f5}=2\varphi_{f4}-\varphi_{f3}$.

The above co- and cross-components of the incident RCP and LCP light coexist under *x*-LP light illumination. On the plane of *z*_1/5_, only the circularly polarized vortex beams of LRCP_A_ and RLCP_B_ components are present, with topological charge of *l*_p, A_ + *l*_g, A_ and *l*_p, B_−*l*_g, B_, respectively. On the plane of *z*_2_, two co-polarized components of the output light field of S_A_ with opposite chiralities and the same topological charge, i.e., RRCP_A_ and LLCP_A_, are present. A linearly polarized vortex beam is formed on this plane due to the superposition of the two components. Similarly, on the plane of *z*_4_, two co-polarized components of S_B_ (i.e., RRCP_B_ and LLCP_B_) are present, thus, another linearly polarized vortex beam is produced. On the plane of *z*_3_, the RLCP_A_ and LRCP_B_ components, with topological charge of *l*_p, A_ − *l*_g, A_ and *l*_p, B_ + *l*_g, B_, are observed. When *l*_p, A_ − *l*_g, A_ = −(*l*_p, B_ + *l*_g, B_), a vector beam is generated.

### 2.3. The Transmitted Light Field of a Single Meta-Atom

The above phase profiles for sub-metasurfaces are designed based on the single meta-atom with the functionality of QWP. For this reason, we analyze the transmitted light field of a single meta-atom in this subsection. A rectangular nanopillar has two vertical mirror symmetry axes in the directions of its length and width. When the nanopillar is illuminated by either linearly polarized light along these axes, the output field experiences different phase delays along each direction. Consequently, form birefringence arises. The above symmetry axes serve as fast and slow axes. Therefore, a rectangular meta-atom can be considered as a waveplate, and its Jones matrix for an arbitrary orientation can be expressed as follows [43]:

(3) $$J(x,y)=R(-\theta )\left[\begin{array}{cc} e^{i\varphi_{x}(x,\;y)} & 0\\ 0 & e^{i\varphi_{y}(x,\;y)} \end{array}\right]R(\theta ),$$

where (*x*, *y*) represents the Cartesian coordinates on the metasurface plane, R(θ) is the rotation matrix, $\varphi_{x}(x,y)$ and $\varphi_{y}(x,y)$ are the phase delays of the output field along the fast and slow axes, respectively. In addition, $\Delta =\varphi_{x}(x,y)-\varphi_{y}(x,y)$ is the phase retardation. $\varphi_{p}=\varphi_{x}$ is the propagation phase imparted on the output light field along the meta-atom waveplate’s fast axis. The approximation of the transmitted amplitudes |*T_xx_*| ≈ |*T_yy_*| ≈ 1 is used in the above equation.

When the QWP meta-atom, with the phase retardation of Δ=π/2, is illuminated with LCP and RCP lights, the transmitted light fields are the superposition of the co- and cross-polarized components with equal amplitudes, which is expressed as follows:

(4) $$J(x,\;y)u^{\sigma =1}=e^{i\varphi_{p}(x,\;y)}u^{\sigma =1}+e^{i\varphi_{\mathrm{LR}}(x,\;y)}u^{-\sigma =-1},$$

(5) $$J(x,\;y)u^{\sigma =-1}=e^{i\varphi_{p}(x,\;y)}u^{\sigma =-1}+e^{i\varphi_{\mathrm{RL}}(x,\;y)}u^{-\sigma =1},$$

where $\varphi_{p}(x,\;y)$ is the phase of the co-polarized component and $\varphi_{\mathrm{LR}/\mathrm{RL}}(x,\;y)$ is the given phase of the corresponding cross-polarized component. Based on the above equations, the propagation phase and orientation angle of the meta-atom can be obtained as follows:

(6) $$\begin{array}{l} \varphi_{x}(x,\;y)=\frac{1}{2}[\varphi_{\mathrm{LR}}(x,\;y)+\varphi_{\mathrm{RL}}(x,\;y)]+\pi \\ \varphi_{y}(x,\;y)=\frac{1}{2}[\varphi_{\mathrm{LR}}(x,\;y)+\varphi_{\mathrm{RL}}(x,\;y)]+\frac{\pi }{2}\\ \theta (x,\;y)=\frac{1}{4}[\varphi_{\mathrm{LR}}(x,\;y)-\varphi_{\mathrm{RL}}(x,\;y)] \end{array}.$$

Substituting Equation (6) into Equations (4) and (5), we can obtain the transmitted light field $E_{t,\;A/B}^{\sigma }(r,\;\beta ,\;0)$ for meta-atom A or B under the illumination of arbitrary circularly polarized (CP) light ***u****^σ^* as follows:

(7) $$E_{t,\;A/B}^{\sigma }(r,\;\beta ,\;0)=J(x,\;y)\cdot u^{\sigma }=\frac{1}{2}e^{i\varphi_{p,\;A/B}}u^{\sigma }+\frac{1}{2}e^{i(\varphi_{p,\;A/B}+\pi /2)}e^{i2\sigma \theta_{A/B}}u^{-\sigma }.$$

For the design of a QWP metasurface, the meta-atom can be chosen from the eight selected meta-atoms based on the propagation phase *φ*_p_, which is closest to the desired phase *φ*_p, A/B_(*r*, *β*, 0), as given in Equation (1). Subsequently, the orientation angles of meta-atoms are determined according to *θ*_A/B_ (*r*, *β*, 0), as given in Equation (2).

By substituting Equations (1) and (2) into Equation (7) and summing the results of *σ* = ±1, the transmitted light field $E_{t,\;A}(r,\;\beta ,\;0)$ with the illumination of *x*-LP light is written as follows:

(8) $$\begin{array}{l} E_{t,\;A}(r,\;\beta ,\;0)=\frac{1}{2}e^{i\pi /2}e^{i\varphi_{f1}}e^{i(l_{p,\;A}+l_{g,\;A})\beta +2i\theta_{0}}\;u^{-\sigma =-1}+\frac{1}{2}e^{i\varphi_{f2}}e^{il_{p,\;A}\beta }(u^{\sigma =1}+u^{\sigma =-1})\\ \;\;\;\;\;+\frac{1}{2}e^{i\pi /2}e^{i\varphi_{f3}}e^{i(l_{p,\;A}-l_{g,\;A})\beta -2i\theta_{0}}\;u^{-\sigma =1} \end{array}.$$

Similarly, under the same condition, the transmitted light field of S_B_, $E_{t,\;B}(r,\;\beta ,\;0)$, is expressed as follows:

(9) $$\begin{array}{l} E_{t,\;B}(r,\;\beta ,\;0)=\frac{1}{2}e^{i\pi /2}e^{i\varphi_{f3}}e^{i(l_{p,\;B}+l_{g,\;B})\beta +2i\theta_{0}}\;u^{-\sigma =-1}+\frac{1}{2}e^{i\varphi_{f4}}e^{il_{p,\;B}\beta }(u^{\sigma =1}+u^{\sigma =-1})\\ \;\;\;\;\;+\frac{1}{2}e^{i\pi /2}e^{i\varphi_{f5}}e^{i(l_{p,\;B}-l_{g,\;B})\beta -2i\theta_{0}}\;u^{-\sigma =1} \end{array}.$$

### 2.4. The Transmitted Light Field of Metasurfaces

**Figure 2:**
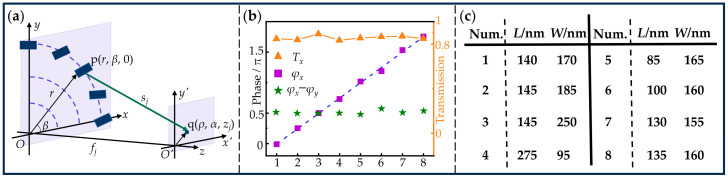
(**a**) Geometry for demonstrating the generation of the longitudinal multi-channel focused beams using QWP metasurface. (**b**) The transmitted amplitudes *T_x_*, propagation phase *φ_x_*, and the phase retardation *φ_x_*–*φ_y_* versus the number of the meta-atoms. (**c**) Dimensions of the selected eight meta-atoms.

We now consider the light field $E(\rho ,\;\alpha ,\;z_{j})$ produced by metasurface S on the observation plane at a distance *z_j_*, which is the sum of $E_{A/B}(\rho ,\;\alpha ,\;z_{j})$ produced by metasurface S_A_ and S_B_. Based on the Rayleigh–Sommerfeld formula, the light field is expressed as follows [44]:

(10) $$E(\rho ,\;\alpha ,\;z_{j})=\frac{if_{j}}{\lambda }\int_{0}^{r_{0}}\int_{0}^{2\pi }[E_{t,\;A}(r,\;\beta ,\;0)+E_{t,\;B}(r,\;\beta ,\;0)]\frac{e^{iks_{j}}}{s_{j}^{2}}rdrd\beta ,$$

where *f_j_* is the focal length of the observation plane, *r*_0_ is the radius of the circular metasurface sample, and *s_j_* is the distance between points p(*r*, *β*, 0) and q(*ρ*, *α*, *z_j_*) on the metasurface and observation planes, respectively, as shown in Figure 2a. Taking *z*_1_ = *f*_1_ as an example, there is only the ***u***^−*σ*=−1^ component of sub-metasurface S_A_, so the light field $E(\rho ,\;\alpha ,\;z_{1})$ on this plane can be expressed as follows:

(11) $$E(\rho ,\;\alpha ,\;z_{1})=\frac{if_{1}}{\lambda }\int_{0}^{r_{0}}\int_{0}^{2\pi }\left[\frac{1}{2}e^{i\pi /2}e^{i\varphi_{f1}}e^{i(l_{p,\;A}+l_{g,\;A})\beta +2i\theta_{0}}u^{-\sigma =-1}\right]\frac{e^{iks_{1}}}{s_{1}^{2}}rdrd\beta .$$

With the approximation condition *f*_1_>>*r*, there is the following:

(12) $$s_{1}\approx \sqrt{f_{1}^{2}+r^{2}}+[\rho^{2}-2r\rho \cos(\alpha -\beta )]/2f_{1}.$$

By substituting Equation (12) into (11), we obtain the following:

(13) $$E(\rho ,\;\alpha ,\;z_{1})=\frac{i}{2\lambda f_{1}}\int_{0}^{r_{0}}\int_{0}^{2\pi }\left[e^{i\pi /2}e^{i\varphi_{f1}}e^{i(l_{p,\;A}+l_{g,\;A})\beta +2i\theta_{0}}\;u^{-\sigma =-1}\right]e^{ik\sqrt{f_{1}^{2}+r^{2}}+ik[\rho^{2}-2r\rho \cos(\alpha -\beta )]/2f_{1}}rdrd\beta .$$

The denominator *s*_1_ in the above equation has been replaced by *f*_1_. By calculating the integral of *β*, we obtain the following:

(14) $$E(\rho ,\;\alpha ,\;z_{1})=C_{1}e^{i\pi /2}e^{i(l_{p,\;A}+l_{g,\;A})\alpha +2i\theta_{0}}\;u^{-\sigma =-1}\int_{0}^{r_{0}}J_{l_{p,\;A}+l_{g,\;A}}(k\rho r/2f_{1})rdr,$$

where $C_{1}=ie^{ik(f_{1}+\rho^{2}/2f_{1})}/2\lambda f_{1}$ and *J_l_*_p, A+*l*g, A_ is the Bessel function of order *l*_p, A_ + *l*_g, A_ of the first kind. Calculating the integral of *r*, Equation (14) can be written as follows:

(15) $$E(\rho ,\;\alpha ,\;z_{1})=C_{1}e^{i\pi /2}e^{i(l_{p,\;A}+l_{g,\;A})\alpha +2i\theta_{0}}F_{h1}(\rho )u^{-\sigma =-1},$$

where *F_h_*_1_(*ρ*) represents the radially variant doughnut of the circularly polarized vortex, which is dependent on the radius of the sample, the topological charge, and the focal length of the light field. Its specific form has been detailed in a previous article [45]. Similarly, the light fields on the other four planes are as follows:

(16) $$E(\rho ,\;\alpha ,\;z_{2})=C_{2}e^{il_{p,\;A}\alpha }F_{h2}(\rho )(u^{\sigma =1}+u^{\sigma =-1}),$$

(17) $$E(\rho ,\;\alpha ,\;z_{3})=C_{3}e^{i\pi /2}F_{h3}(\rho )[e^{i(l_{p,\;A}-l_{g,\;A})\alpha -2i\theta_{0}}\;u^{-\sigma =1}+e^{i(l_{p,\;B}+l_{g,\;B})\alpha +2i\theta_{0}}\;u^{-\sigma =-1}],$$

(18) $$E(\rho ,\;\alpha ,\;z_{4})=C_{4}e^{il_{p,\;B}\alpha }F_{h4}(\rho )(u^{\sigma =1}+u^{\sigma =-1}),$$

(19) $$E(\rho ,\;\alpha ,\;z_{5})=C_{5}e^{i\pi /2}e^{i(l_{p,\;B}-l_{g,\;B})\alpha -2i\theta_{0}}F_{h5}(\rho )u^{-\sigma =1}.$$

Compared with C_1_, the components of C_2_, C_3_, C_4_, and C_5_ can be obtained by replacing *f*_1_ with *f*_2_, *f*_3_, *f*_4_, and *f*_5_, respectively. Equations (15)–(19) obviously illustrate the propagation distances, topological charges, and chiralities of these vortices, and they also demonstrate that two circularly polarized vortices, two linearly polarized vortex beams, and one vector beam are generated on five distinct focal planes.

### 2.5. Generation of Focused HOP Beams with Tunable Elliptically Polarized Light Illumination

Next, we consider the transmitted light field on the plane of *z*_3_ under the illumination of tunable elliptical polarization. The incident light is the superposition of the RCP and LCP beams with unequal weights, as follows:

(20) $$E_{\mathrm{in}}=\cos(\Theta /2)e^{i\Phi /2}u^{\sigma =-1}+\sin(\Theta /2)e^{-i\Phi /2}u^{\sigma =1},$$

where (***Θ***, ***Φ***) represents the spherical coordinate on the conventional Poincaré sphere (PS) and cos(***Θ***/2) and sin(***Θ***/2) are the amplitudes of the RCP and LCP components, respectively. Based on Equation (17), the components $u^{-\sigma =-1}$ and $u^{-\sigma =1}$ originate in components $u^{\sigma =1}$ and $u^{\sigma =-1}$ of the incident light. Therefore, the HOP beams generated by the QWP metasurface are expressed as follows:

(21) $$E(\rho ,\;\alpha ,\;z_{3})=C_{3}e^{i\pi /2}F_{h3}(\rho )[\sin(\Theta \prime /2)e^{i\Phi \prime /2}u^{-\sigma =-1}+\cos(\Theta \prime /2)e^{-i\Phi \prime /2}u^{-\sigma =1}].$$

Here, ***Θ****’* and ***Φ****’* represent the latitude and longitude of VBs on the HOP sphere, and the relationships of (***Θ***, ***Φ***) and (***Θ****’*, ***Φ****’*) are $\Phi \prime =4\theta_{0}-\Phi$ and Θ′=π−Θ, respectivley. For *x*-LP light illumination with ***Φ*** = 0, taking 4*θ*_0_ = 0, *π*/2, *π*, and 3*π*/2, the generated vector beams are the familiar VBs of radial, 45°-slanted, azimuthal, and 135°-slanted polarizations, respectively. In this study, the azimuthal vector beam is generated on the plane of *z*_3_.

## 3. Simulation Results

### 3.1. Simulation of the Transmitted Light Field of the Meta-Atom

To demonstrate the proposed design, we simulated the transmitted light field using the FDTD method. This simulation contains two parts. The first one is running two-dimensional parametric sweeps with the side lengths of the nanopillar as the parameters for selecting the desired eight QWP meta-atoms. The second one is simulating the transmitted light field of the designed metasurface. We first describe the process of parametric sweeps, which is for a single meta-atom. The dielectric material of the rectangular nanopillar was a-Si:H, with the refractive index of *n* = 3.744 and extinction coefficient of *κ* = 0.000 at the wavelength of *λ* = 800 nm, while the substrate material was fused silica. The height *H* and lattice period *P* of the meta-atom were 480 nm and 380 nm, respectively. The side length ranged from 80 nm to 330 nm in steps of 1 nm. In the simulation, the maximum mesh step was 10 nm. Under the illumination of *x*-polarized light, the corresponding boundary conditions for *x*, *y*, and *z* were “Anti-symmetric”, “Symmetric”, and “Perfectly Matched Layer (PML)”, respectively. Similarly, the corresponding boundary conditions for *x*, *y*, and *z* were “Symmetric”, “Anti-symmetric”, and “Perfectly Matched Layer (PML)”, respectively, with *y*-polarized light illumination. Consequently, the transmitted amplitudes and propagation phases as functions of the side lengths were obtained. Eight QWP meta-atoms were selected under the conditions that the propagation phase $\varphi_{x}$ increased uniformly from 0 to 2*π* and the phase retardation $\Delta =\varphi_{x}-\varphi_{y}$ was equal to *π*/2. Accordingly, these selected meta-atoms were labeled sequentially from one to eight. Figure 2b shows the transmitted amplitudes *T_x_*, the propagation phase $\varphi_{x}$, and the phase retardation $\Delta =\varphi_{x}-\varphi_{y}$ of eight meta-atoms. The blue dashed line is the linear fit of $\varphi_{x}$. The results are consistent with the above conditions for selecting meta-atoms. The dimensions of these meta-atoms are shown in Figure 2c.

### 3.2. Simulation of the Longitudinally Multi-Channel Light Field Generated by the Metasurface

Then, a metasurface sample was designed using the selected eight meta-atoms. The sample had a diameter of 114 μm, with focal lengths of *f*_1_ = 30 μm, *f*_2_ = 40 μm, *f*_3_ = 52 μm, *f*_4_ = 65 μm, and *f*_5_ = 82 μm. The topological charges were set as *l*_p, A_ = 1, *l*_g, A_ = 2, *l*_p, B_ = −1, and *l*_g, B_ = 2. Subsequently, we simulated the transmitted light field of the entire metasurface sample. In the simulation, the corresponding boundary conditions for *x*, *y*, and *z* were all PML. The position of the substrate of the metasurface was set to *z* = 0, and the incident light was set to *z* = −0.8 μm. The electric field monitor, set at z = 1 um, captured the simulated light field data and stored the results to a file for subsequent analysis. The region of FDTD computation, which covers the range of the incident light, metasurface and monitor, was 116 μm × 116 μm × 2.2 μm, where the z span was set from −1 μm to 1.2 μm. A non-uniform mesh was automatically configured in a self-adaptive manner in the FDTD software with the mesh accuracy set as level 1, the number of mesh points reported by the software was 3354 × 3354 × 37 (= 4.2 × 10^8^), and the reported minimum mesh step was 10 nm. The memory assumption of the running simulation reported by the software was 35.679 GB. The simulation was performed with the Dell workstation (DESKTOP-9LKPEBS) and Windows 10 with the memory extension set to 256 GB in several hours. Using the near-to-far projection functions in the software, the near-field data were projected to the desired far-field on the observation planes. The near-to-far field projection functions, offered by the FDTD software (Lumerical 2020 R2), enabled efficient and accurate calculation of electromagnetic fields in the far-field using near-field data and plane wave decomposition. Comparing the above processes of simulation, parametric sweep consumes relatively little memory, but it is time-consuming; calculating near-field data consumes memory but takes relatively little time; and near-to-far projection consumes neither memory nor time, and it is an efficient method for calculating light field in far-field regions. In addition, it should be noted here that *z* = 0 was the coordinate set for the FDTD simulation, which is slightly different from *z* = 0 at the height of the nanopillar used in the above theoretical analysis.

**Figure 3:**
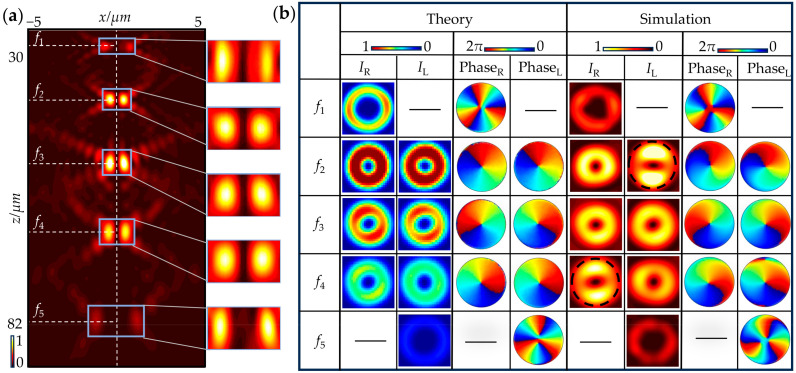
Theoretical and simulation results of longitudinal multi-channel tunable focused vortex and vector beams. (**a**) Simulated total intensity image of these beams under *x*-LP light illumination in the *x*-*z* plane, along with corresponding enlarged views. (**b**) Theoretical and simulated intensity and phase images of these beams on each focal plane.

Figure 3a shows the simulated total intensity image of the longitudinal multi-channel tunable focused vortex and vector beams with *x*-LP light illumination in the *x*-*z* plane. The positions of the five focal planes are framed in the figure, with enlarged views provided in the right panel. Notably, in order to observe the shape of each focal point, the enlarged views are shown with different color scales. Figure 3b shows both theoretical and simulated intensity images on each slice. The left title column shows the positions of five focal planes, denoted as *f_j_*. The first three rows present the corresponding intensity and phase labels of the vortex and vector beams in theory and simulation. In addition, the vortices are encircled by dashed lines to facilitate the characterizations of the corresponding vortices. All vortices exhibit doughnut-shaped intensity distribution. On the planes of *z*_1/5_, only the intensity and phase images of the single existing CP vortex are given, with the order of ±3, while the orders of other vortices are ±1. Based on these results, it is obvious that the simulation results are consistent with the theoretical analyses. However, some discrepancies remain, including the non-uniform distributions of the total and component field intensities of the vortex beams, as well as a slight inclination in the lobes of the vector beam. These deviations can be attributed to two primary factors. First, the selected eight meta-atoms are not ideal QWPs, introducing deviations in propagation phase and transmission amplitude, which result in non-uniform contributions on the superposed focal plane. Second, the multi-focal design inherently introduces cross-talk between the focal planes, where diverged waves from adjacent focal planes induce non-paraxial interference patterns and diffractive coupling, leading to imperfections in intensity distributions.

**Figure 4:**
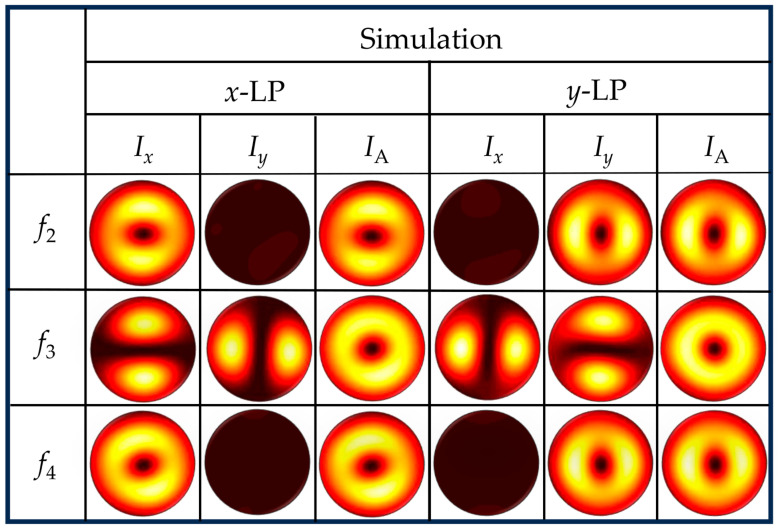
The simulation results of the vortex and vector beams on the planes of *z_j_*, *j* = 2, 3, and 4, under the illumination of *x*- and *y*-LP light, respectively. The left title column shows the propagation distances of the observation planes. The first and second title rows show the polarization direction of the incident light and the components of the output light, respectively.

In order to examine the distribution characteristics of the beams on the planes of *z_j_*, where *j* = 2, 3, and 4, the simulated intensity images with *x*- and *y*-LP light illuminations are given in Figure 4. The left column indicates the distances of the observation planes. The first title row indicates the polarized direction of the incident light, while the second title row specifies the *x*-, *y*-component and total intensities, respectively. The intensity distributions of the linearly polarized vortex beams on the planes of *z*_2/4_ show distinct component patterns under different linearly polarized light illumination. In Figure 4, the *x*-component intensity *I_x_* = |***E****_x_*|^2^ and total intensity *I*_A_ = *I_x_* + *I_y_* =|***E****_x_*|^2^ + |***E****_y_*|^2^ images both show the doughnut-shaped intensity distributions, whereas the *y*-component intensity *I_y_* = |***E****_y_*|^2^ disappears under *x*-LP light illumination. Conversely, under *y*-LP light illumination, the *I_y_* and *I*_A_ images both show doughnut-shaped intensity distributions, whereas *I_x_* disappears. This indicates that the polarized direction of the linearly polarized vortex beam is related to the illumination light. For the vector beam of azimuthal polarization on the plane of *z*_3_, *I_x_* and *I_y_* show two vertical and horizontal lobes, respectively, and *I*_A_ shows doughnut-shaped intensity distribution with *x*-LP light illumination. With *y*-LP light illumination, *I_x_* and *I_y_* of the vector beam show two horizontal and vertical lobes, respectively, while *I*_A_ maintains doughnut-shaped intensity distribution, indicating that it is a vector beam of radial polarization. While a non-negligible longitudinal field component *I*_z_ persists in the focal planes, our previous theoretical and experimental studies [46,47] confirm its negligible impact on transverse intensity measurements of *I*_x_ and *I*_y_. This is attributed to the fact that *E_z_* propagates predominantly in the *x*-*y* plane and perpendicular to the *z*-axis, rendering *I_z_* unobservable when the observation plane coincides with the *x*-*y* plane. Consequently, the *I_z_* was intentionally omitted from the simulation results, which focus on the dominant in-plane *I*_x_ and *I*_y_ components. These results demonstrate that the polarization states of light fields, formed through vortex superposition in relation to the incident light, can be effectively generated by our sample.

### 3.3. Simulation of the HOP Beams Produced by the Metasurface

**Figure 5:**
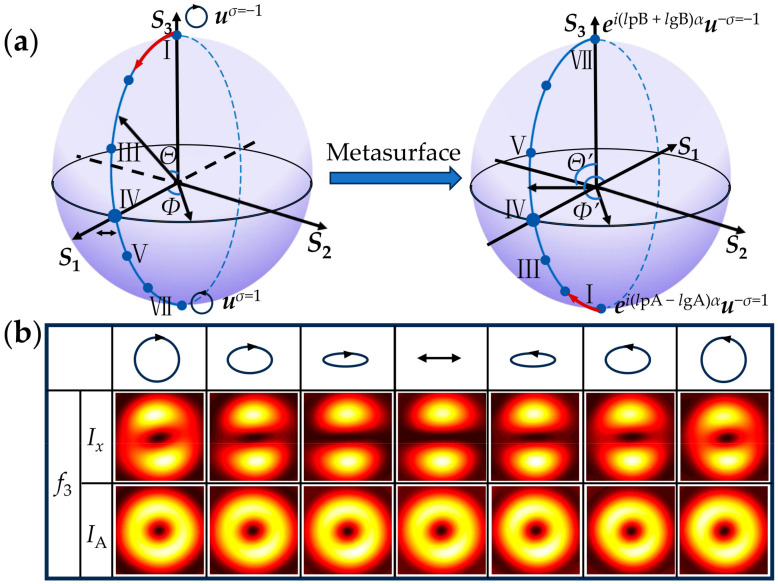
(**a**) The transformation of the polarization states on the conventional PS to HOP sphere by the metasurface. The Roman numbers I–VII represent the polarization states on a meridian. (**b**) Simulated *x*-component and total intensity patterns, *I_x_* and *I*_A_, of the VBs on the plane of *z*_3_ under the elliptically polarized light illumination. The black double arrow, elliptical arrows, and round arrows represent the incident polarizations.

In Section 2.5, the generation of focused HOP beams on the plane of *z*_3_ with the incident light of elliptical polarization states was analyzed. Figure 5 shows the corresponding simulation results. Figure 5a depicts the transformation of the polarization states from the conventional PS to HOP sphere by the metasurface. The elliptical polarization states of the incident light with spherical coordinates (*Θ*, *Φ*)_PS_ were transformed into HOP beams according to the mapping relation (*Θ*, *Φ*)_PS_ → (*Θ*′ = *π* − *Θ*, *Φ*′ = *π* − *Φ*)_HOPS_. Figure 5b shows the simulated *x*-component and total intensity images, *I_x_* and *I*_A_, of the HOP beams. The left columns show the propagation distance of the observation plane, along with the corresponding *x*-component and total intensity images. The black double arrow, elliptical arrows, and round arrows in the top row present the polarization of the incident light. From left to right, the corresponding polarization states are represented by seven points, I–VII, which uniformly divide the longitudinal line of *Φ* = 0° from the north to south poles on the conventional PS. These results indicate that, when the incident polarization state evolves from the north to south poles via the equator along the longitudinal line of *Φ* on the conventional PS, the VBs move symmetrically in the opposite direction from the south to north poles via the equator along the longitudinal line of *Φ*′ = *π* − *Φ* on the HOP sphere. In addition, the *x*-component intensity image changes gradually from a doughnut to a distinct two-lobe pattern, and then gradually blurs until it evolves as a vortex doughnut at the north pole, with incident polarization states varying.

## 4. Discussion and Conclusions

The spin-dependent geometric phases, with equal and opposite signs, cause the conjugated symmetry of the transmitted wavefields under the illuminations of CP beams, resulting in the so-called spin-locked effect for LCP and RCP light. The functionalities of geometric metasurfaces with identical dimension meta-atoms are often locked and mirrored for the two CP beams owing to spin locking. Previous studies have demonstrated the generation of the transverse multi-channel focused vector beams using HWP metasurfaces with the designed geometric hyperbolic phase profiles. However, the incident beams are split into convergent and divergent components because of the spin-locked effect [48]. To extend the functionalities, the spin-decoupled metasurfaces capable of independently manipulating the two orthogonal CP components (*x*- and *y*-LP light) have been developed. Combing the propagation and geometric phases, the co- and cross-components can be simultaneously controlled, enabling the generation of multi-channel focused vortex and vector beams using spin-decoupled QWP metasurfaces, thereby providing more flexibility for optical field manipulation. In addition, the observed multi-focal behavior results from the designed phase profiles, but it is also influenced by practical conditions. Periodical meta-atoms with spatially varying dimensions and orientation angles may affect ideal beam generation due to the diffraction and scattering effects. Meanwhile, the inherent discreteness of the meta-atom phase response may introduce residual phase errors between the ideal and real phase profiles of sub-metasurfaces. These factors collectively contribute to deviations from the ideal multi-focal behavior, while our metasurface design still preserves the overall intended functionality.

In this study, we designed a QWP metasurface to generate longitudinally multi-channel focused vortex and vector beams. The metasurface comprises two interleaved sub-metasurfaces. The helical phase profiles are designed independently in the propagation and geometric phases of each sub-metasurface to generate vortices in co- and cross-polarized components with corresponding topological charges. Subsequently, the hyperbolic phases are designed in the propagation and geometric phases of each sub-metasurface for focusing the co- and cross-polarized components to the pre-set positions. Under the illumination of *x*-LP light, on the plane of *z*_1/5_, an RCP/LCP vortex with topological charge *l*_p, A/B_ ± *l*_g, A/B_ is observed. On the plane of *z*_2/4_, two orthogonal circularly polarized vortices, with the same topological charge *l*_p, A/B_, are formed. On the plane of *z*_3_, two orthogonal circularly polarized vortices, with topological charge *l*_p, A/B_ ∓ *l*_g, A/B_, are generated, resulting in a vector beam with *l*_p, A_ − *l*_g, A_ = −(*l*_p, B_ + *l*_g, B_). Consequently, two circularly polarized vortices, two linearly polarized vortices, and one vector beam are generated on five focal planes. In addition, vortex and vector beams with different linear polarizations, as well as HOP beams on a meridian of a HOP sphere, are also produced by adjusting the polarization of the incident light. Our study, which explores the manipulation of the vortex and vector beams along the propagation distance, promotes the development of integrated and multifunctional optical devices and systems. The findings hold significant potential applications in light–matter interaction, laser processing, and optical communication.

## Data Availability

The data that support the findings of this study are available from the corresponding author upon reasonable request.
